# Ultrasound-guided radiofrequency ablation versus laparoscopic adrenalectomy for unilateral isolated aldosterone-producing adenoma: a two-center retrospective study

**DOI:** 10.3389/fendo.2026.1880658

**Published:** 2026-09-07

**Authors:** Lingpeng Tang, Bingzi Zou, Kai Li, Dandan Wang, Xiaoying Lin, Ting Hu, Jianchuan Yang, Songsong Wu

**Affiliations:** 1 Shengli Clinical Medical College of Fujian Medical University, Fujian Medical University, Fuzhou, China; 2 Department of Ultrasonography, Fujian Provincial Hospital, Fuzhou University Affiliated Provincial Hospital, Fuzhou, China; 3 Department of Ultrasonography, Huizhou Central People’s Hospital, Huizhou, China; 4 Department of Ultrasound, The Third Affiliated Hospital of Sun Yat-sen University, Guangzhou, China; 5 Department of Ultrasonography, The Second Affiliated Hospital of Fujian Medical University, Quanzhou, China

**Keywords:** aldosterone-producing adenoma, hypertension control, laparoscopic adrenalectomy, minimally invasive treatment, ultrasound-guided radiofrequency ablation

## Abstract

**Objective:**

This study aimed to evaluate the safety and efficacy of Ultrasound-guided radiofrequency ablation (US-guided RFA) compared to laparoscopic adrenalectomy (LA) for the treatment of aldosterone-producing adenoma (APA).

**Methods:**

This retrospective, two-center study evaluated 100 patients with APA treated with either US-guided RFA or LA between January 2020 and June 2024. Patients were categorized based on treatment modality (US-guided RFA: n=36; LA: n=64). To minimize confounding, a 1:1 matching was performed based on sex, age, medical history, and disease characteristics. Post-treatment outcomes, including biochemical remission and blood pressure control, were compared using independent-samples t-tests or chi-square tests.

**Results:**

A total of 36 patients (median age: 49.5 years [IQR: 33.25–57.00]; 23 women) underwent US-guided RFA, and 36 matched patients (median age: 52.00 years [IQR: 37.50–59.75]; 20 women) underwent LA. The technical success rate was 100% in both groups. During the follow-up period (median: 14 months [IQR: 6–22 months]), both groups achieved complete biochemical remission (36/36, *P* = 1.000), similar hypertension control rates (66.7% vs. 77.8%, *P* = 0.430), and no disease progression (0/36 in both groups). US-guided RFA was associated with significantly lower intraoperative blood loss (median: 3.0 mL vs. 30 mL) and shorter procedure time (77.5 min vs. 115 min; both *P* < 0.001).

**Conclusion:**

In patients with unilateral isolated APA, no significant differences were observed between US-guided RFA and LA in terms of disease progression, biochemical remission, or hypertension control in the short term.

## 1 Introduction

Aldosterone-producing adenoma (APA) is a major etiological factor underlying primary aldosteronism (PA) (1). Excessive aldosterone secretion by APA can lead to metabolic disorders such as treatment-resistant hypertension and hypokalemia, significantly increasing the risk of cardiovascular and renal target organ damage (2, 3). Currently, laparoscopic adrenalectomy (LA) is the first-line treatment recommended by clinical guidelines for APA management (4). However, not all patients are willing or suitable to undergo surgery (5). Although pharmacological treatment can partially manage hypertension and hypokalemia, long-term medication use is associated with dose-dependent adverse effects and poor patient compliance (6, 7). Therefore, minimally invasive interventional strategies warrant further investigation as potential treatment options for patients with APA who are considered unsuitable for surgery after comprehensive evaluation or who decline surgery after being fully informed.

In recent years, minimally invasive interventional techniques have garnered increasing attention for APA treatment. These include transcatheter ablation (8), endoscopic ultrasound-guided ablation (9), and CT-guided radiofrequency ablation (10). However, these methods are often limited by stringent patient selection criteria, procedural complexity, and the risk of incomplete ablation. With the advancement of percutaneous ultrasound-guided technology, this modality has demonstrated efficacy in treating adrenal metastases (11). Compared with CT guidance, the US-guided technique provides real-time dynamic imaging and repeated ultrasonography (12, 13). These advantages significantly improve lesion localization accuracy, particularly for microtumors that may be missed by CT. Additionally, the intraoperative use of multimodal ultrasound technology enables real-time monitoring of the ablation boundary and thermal diffusion (14). This reduces the risk of tumor remnants and minimizes complications such as adrenal hematomas and thermal injuries to adjacent organs. These benefits provide both theoretical justification and practical support for applying percutaneous ultrasound-guided radiofrequency ablation (US-guided RFA) of APA.

Despite the promising potential of US-guided RFA for APA treatment, there remains a lack of systematic comparisons with LA. This study aims to compare the therapeutic efficacy and safety profiles of US-guided RFA and LA in APA patients. Specifically, we assess differences in treatment effectiveness, disease progression, and complication rates to inform clinical decision-making regarding optimal treatment strategies.

## 2 Materials and methods

### 2.1 Patients and study design

This retrospective, two-center study was approved by the Human Ethics Review Committee of the Provincial Hospital of Fuzhou University and the Third Hospital of Sun Yat-sen University (Approval No. K2024-12-015). Clinical records of patients with adrenal lesions who were evaluated for US-guided RFA or LA between January 2020 and June 2024 were reviewed. All patients provided written informed consent for treatment, and the requirement to obtain informed consent for study inclusion was waived because the personal details of these patients were kept confidential. Some US-guided RFA cases overlap with a prior submission; this study expands the sample, adds LA comparison, and evaluates operative outcomes for treatment selection.

US-guided RFA was performed in patients with clinically confirmed unilateral APA who declined surgery or had an inadequate response to medical therapy. The inclusion criteria were as follows: (1) ARR >30 (ng/dL)/[μg/(L·h)]; (2) biochemically confirmed PA, with unilateral disease assessed by adrenal CT together with adrenal venous sampling (AVS) and/or adjunctive C-X-C chemokine receptor type 4 (CXCR4)-targeted nuclear imaging; (3) tumor diameter <4 cm, clear visualization of the lesion on preoperative ultrasonography, and availability of a safe needle path for US-guided RFA; (4) presence of treatment-resistant hypertension or hypertension with hypokalemia. Exclusion criteria included: (1) severe cardiopulmonary dysfunction precluding surgery; (2) malignancy, hepatic or renal insufficiency, or other serious comorbidities; (3) imaging findings suggestive of bilateral adrenal disease, multiple adrenal lesions, or other concomitant adrenal disorders; (4) pathologically confirmed adrenal metastases; (5) explicit refusal to participate in the study; and (6) incomplete follow-up data required for the minimum 6-month clinical and biochemical outcome assessment. Biochemical confirmation of PA was based on plasma aldosterone concentration, plasma renin activity, the ARR, and, where indicated, saline infusion testing. For the saline infusion test, 2 L of 0.9% saline was administered over 4 hours, and a post-infusion plasma aldosterone concentration >10 ng/dL was considered indicative of PA (15). Subtype evaluation comprised adrenal CT and AVS, with CXCR4-targeted nuclear imaging used as an adjunctive localization method in selected patients. AVS was performed with or without adrenocorticotropic hormone (ACTH) stimulation. The selectivity index was calculated as the adrenal-vein-to-inferior-vena-cava cortisol ratio; bilateral adrenal-vein catheterization was considered successful when the selectivity index was ≥2 without ACTH stimulation or ≥5 with ACTH stimulation. The lateralization index was calculated as the dominant-to-contralateral adrenal-vein aldosterone-to-cortisol ratio, and a value ≥4 indicated unilateral aldosterone secretion (15, 16).

### 2.2 Pretreatment assessment

All patients with APA scheduled for LA or US-guided RFA underwent thorough preoperative assessment to optimize their physical condition. Pre-treatment goals included achieving adequate blood pressure control and correcting any hypokalemia to ensure favorable conditions for intervention. Prior to treatment, patients underwent routine ultrasonography, contrast-enhanced ultrasound (CEUS), abdominal CT, and laboratory testing related to PA.

Ultrasound examinations were performed using an EPIQ 7 ultrasound system (Philips, The Netherlands) equipped with a 2.0–5.0 MHz broadband convex array transducer for lesion evaluation and procedural guidance. Conventional ultrasound was used to assess tumor size (measuring three orthogonal diameters), location, and sonographic features. Tumor volume was calculated using the ellipsoid formula: V = πabc/6, where a is the largest transverse diameter, and b and c are vertical diameters (17).

CEUS was performed to characterize lesion vascularity and improve sonographic delineation of the target nodule. A 2.4 mL bolus of SonoVue (Bracco Imaging, Italy) was injected into the median cubital vein, followed by a 5 mL saline flush. Tumor enhancement was evaluated relative to the adjacent normal tissue and categorized as hypoenhancement, isoenhancement, or hyperenhancement. Autonomous aldosterone secretion was confirmed by biochemical evaluation, including the saline infusion test, whereas unilateral functional localization was assessed by AVS, with CXCR4-targeted nuclear imaging used as an adjunctive localization method in selected patients. For RFA planning, the sonographically identified target was correlated with the adrenal lesion demonstrated on cross-sectional imaging and the available functional localization findings. CEUS findings were incorporated into this multimodal assessment to support lesion targeting and procedural planning.

Puncture samples were analyzed histopathologically or cytologically to provide additional diagnostic support for treatment planning. Laboratory evaluations included serum aldosterone, plasma renin activity, blood potassium, and ARR, among other relevant biomarkers.

### 2.3 Treatment

#### 2.3.1 Laparoscopic adrenalectomy

LA was performed by two urologists, each with over 10 years of surgical experience. Patients were positioned laterally, and the choice between a transperitoneal or retroperitoneal approach was determined based on tumor location. The retroperitoneal approach typically employed the jackknife position with lumbar padding to fully expose the operative field. A pneumoperitoneum was established (intra-abdominal pressure: 12–15 mmHg) via abdominal or lumbar puncture, followed by the placement of 3–4 trocars (sizes: 5 mm, 10 mm, and 12 mm).

In the transperitoneal approach, the lateral peritoneum was incised, and adjacent structures such as the colon, spleen, or liver were mobilized to visualize the adrenal region. In contrast, the retroperitoneal approach involved direct dissection of retroperitoneal fat to access the periadrenal space. A combination of blunt and sharp dissection was used to separate periadrenal fat while protecting surrounding organs. Tumors were localized based on preoperative imaging and carefully mobilized along the peritoneum to avoid rupture.

Depending on the size and pathology of the tumor, either partial or total adrenalectomy was performed. The central adrenal vein was ligated using arterial clips and dissected using an ultrasonic scalpel or electrocautery hook. After resection, meticulous hemostasis was ensured, and the specimen was placed in a retrieval bag and extracted through a trocar site. Drains were placed if necessary. The pneumoperitoneum was then evacuated, and trocar sites were closed.

Throughout the procedure, careful handling was essential to minimize bleeding and avoid injury to adjacent structures (e.g., inferior vena cava, duodenum, splenic vessels). Following LA, patients received routine postoperative care and were typically discharged 2–3 days after surgery.

#### 2.3.2 Ultrasound-guided radiofrequency ablation

Prior to ablation, multimodal imaging, including conventional ultrasound and CEUS (sulfur hexafluoride microbubbles, SonoVue; Bracco) was performed to localize the adrenal lesion. US-guided RFA was performed by two radiologists and one anesthesiologist, each with more than 7 years of clinical experience. The procedure was carried out with the patient in the supine or lateral position, selected to optimize the puncture route according to lesion location.

Local anesthesia with 1% lidocaine was administered at the puncture site, and basal intravenous anesthesia was maintained throughout the procedure. An 18-gauge core needle was inserted under ultrasound guidance (Curaway CRS2000) for hydrodissection. A slow infusion of 200–400 mL of 5% dextrose solution was then administered. This isolated the lesion from adjacent critical structures, including the inferior vena cava, liver, kidneys, colon, and spleen. A minimum safety margin of 2 cm was maintained.

A 16G coaxial needle and 18G biopsy needle were used to obtain histopathological samples. A 17-gauge unipolar radiofrequency ablation electrode (20-mm active tip; Curaway CRS2000) was then inserted through the coaxial sheath. Ablation was performed at a power setting of 30–100 W, using a fixed and mobile technique depending on tumor characteristics. Deeper and perivascular regions were ablated first, followed by ablation from deep to superficial layers. Each ablation point was maintained for 120 seconds, and the hyperechoic ablation zone was extended at least 2 mm beyond the tumor margin.

CEUS was performed 5 minutes after the procedure to evaluate the ablation effect. Additional ablation was conducted immediately in cases where residual enhancement was observed or if the ablation margin was insufficient. In patients with small, deep, or poorly visualized lesions, fusion imaging with the Esaote MyLab Class C system and Virtual Navigator software was used for accurate guidance (left vs. right = 17:19). Manual pressure was applied to the puncture site for 5 minutes post-procedure, followed by a 30-minute observation to monitor for bleeding. Following ablation, patients were closely monitored for 48 hours, including assessment of vital signs, PA-related biochemical parameters, and procedure-related complications.

**Figure 1:**
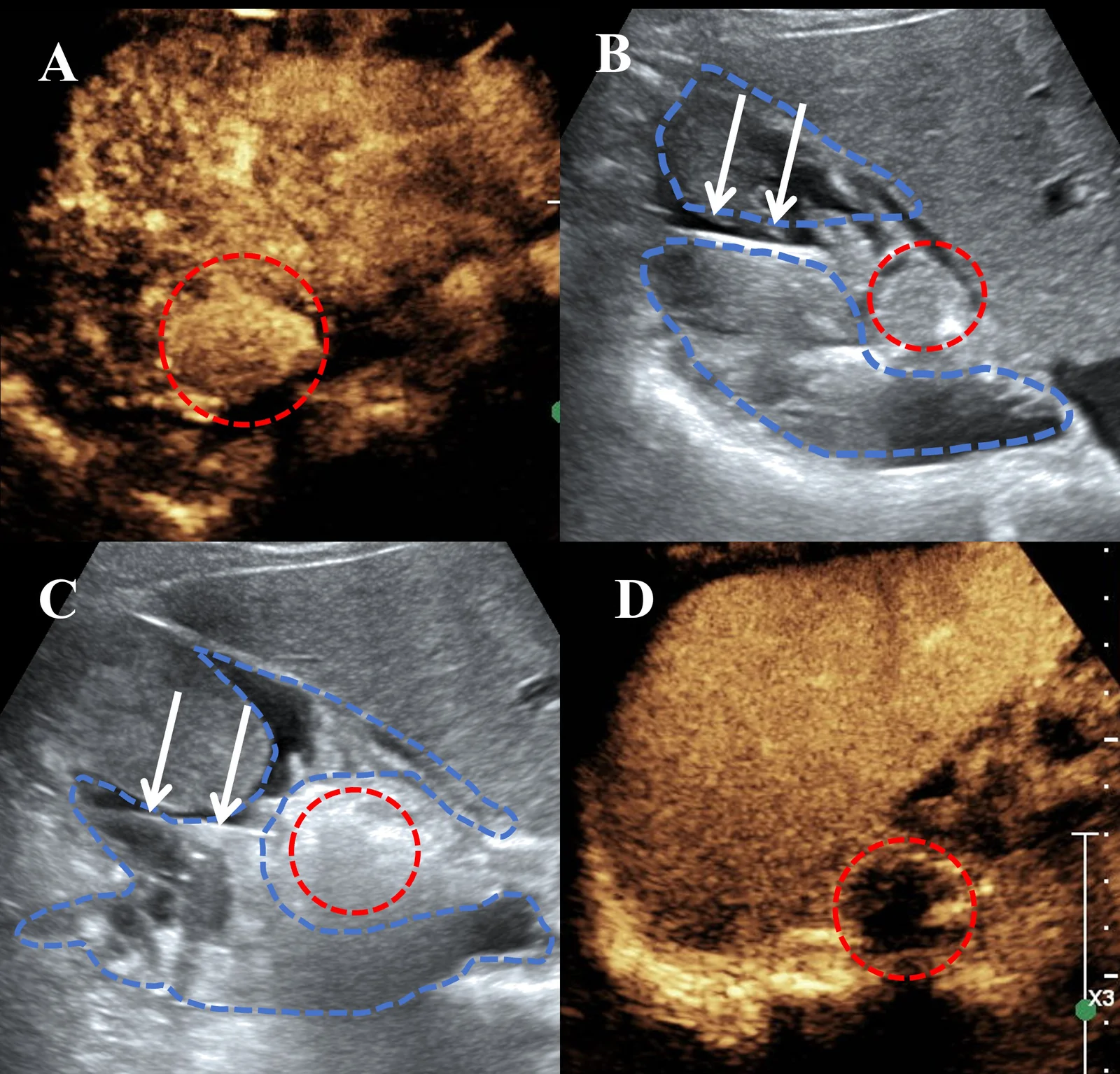
US-guided RFA in a 56-year-old female patient with right-sided aldosterone-producing adenoma. **(A)** Pre-ablation CEUS displays a hyperenhancement pattern in the APA (circle). **(B)** Hydrodissection technique applied to protect surrounding vital organs (dotted line). **(C)** Intraoperative ultrasound provides real-time monitoring of the RFA procedure (circle). **(D)** Post-ablation CEUS shows no contrast enhancement within the ablation zone (circle). APA, aldosterone-producing adenoma; US-guided RFA, ultrasound-guided radiofrequency ablation; CEUS, contrast-enhanced ultrasound.

Vital signs were continuously monitored by the anesthesiologist. Intraoperative severe hypertension was defined as a transient increase in systolic blood pressure to ≥180 mmHg and/or diastolic blood pressure to ≥120 mmHg during the procedure. Intravenous labetalol (20 mg per dose) was administered when indicated to maintain a target blood pressure of <140/90 mmHg. Representative images of the ablation process are shown in **Figure 1**.

### 2.4 Follow-up

Clinical data were systematically recorded, including patient demographics, tumor characteristics, procedure duration, length of hospital stay, and estimated blood loss. Length of hospital stay was defined as the period from admission to discharge. For patients who completed biochemical confirmation of PA or subtype evaluation during the same admission before treatment, the corresponding pretreatment period was included. Follow-up assessments were conducted on the day after treatment, at 1 week, 1 month, and 3 months, and subsequently every 6 months. At each follow-up visit, CEUS and relevant laboratory tests were performed. Laboratory evaluations included plasma ARR and serum potassium levels to monitor PA control and correction of hypokalemia. CEUS was used to assess complete ablation of the targeted lesion.

Treatment-related complications were recorded according to their type and severity and were graded according to the Clavien–Dindo classification.

### 2.5 Definition of disease progression and technical success

Technical success of US-guided RFA was defined as the complete absence of enhancement in the treated lesion on CEUS immediately after ablation, whereas technical success of LA was defined as successful resection of the target adrenal lesion, supported by histopathological confirmation of an APA. In both groups, biochemical remission was defined as normalization of the ARR at the final follow-up. Clinical success was defined as either achievement of blood pressure control with antihypertensive medication or a sustained reduction in blood pressure from baseline during follow-up.

Persistent PA was defined as an abnormal ARR at or beyond the 3-month follow-up. Recurrence was defined as normalization of ARR at 3 months followed by a subsequent elevation at later follow-up. LA was considered in cases of persistent or recurrent PA with uncontrolled blood pressure.

If persistent PA or incomplete ablation was identified after the initial US-guided RFA session, a second RFA session was considered. For patients with inadequate disease control after two RFA sessions, alternative treatment, including LA, was considered.

### 2.6 Statistical analysis

Continuous data are expressed as mean ± standard deviation if they follow a normal distribution, or as median (interquartile range, IQR) if they do not. Categorical variables are presented as frequencies (percentages). Patient characteristics, tumor features, and post-treatment outcomes were compared between groups. Relative changes in blood pressure and biochemical parameters were calculated as (pretreatment value − post-treatment value)/pretreatment value. The Mann-Whitney U test was used for non-normally distributed continuous data. The independent samples t-test was used for normally distributed continuous data. Categorical variables were analyzed using the chi-square test or Fisher’s exact test. A 1:1 matching method was employed to minimize the impact of confounding factors. All tests were two-sided, and a *P*-value < 0.05 was considered statistically significant. Statistical analyses were performed using SPSS software (version 26.0; IBM).

## 3 Results

### 3.1 Demographic characteristics

This retrospective study screened 168 patients with adrenal lesions who were evaluated at the two participating centers between January 2020 and June 2024. Sixty-eight patients were excluded: 13 with multiple adrenal lesions (US-guided RFA, n = 4; LA, n = 9), 26 with adrenal metastases (US-guided RFA, n = 8; LA, n = 18), 18 with no biochemical evidence of PA (US-guided RFA, n = 8; LA, n = 10), and 11 with incomplete 6-month follow-up data (US-guided RFA, n = 7; LA, n = 4). The final cohort comprised 100 patients with unilateral isolated APA. The mean age was 49.81 ± 12.35 years, with a male-to-female ratio of 51:49.

**Figure 2:**
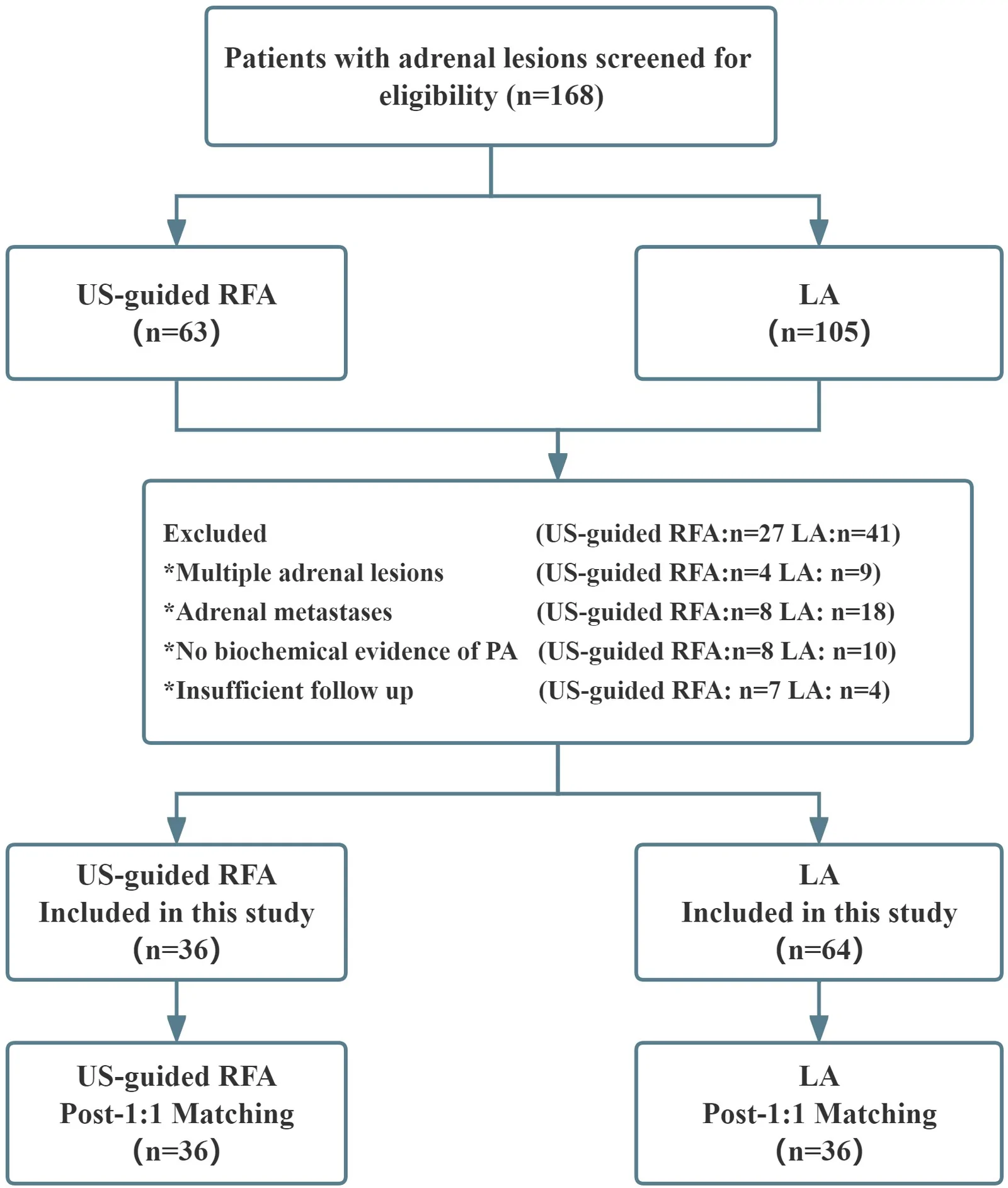
Patient flowchart. US-guided RFA, ultrasound-guided radiofrequency ablation; LA, laparoscopic adrenalectomy; APA, aldosterone-producing adenoma.

Patients were initially divided into a US-guided RFA group (n = 36) and a LA group (n = 64) based on treatment modality. After 1:1 matching for sex, age, medical history, and disease characteristics, each group included 36 patients (US-guided RFA: n = 36; LA: n = 36) (**Figure 2**).

There were no significant differences in matched baseline characteristics between the two groups, except for treatment cost, which was significantly lower in the US-guided RFA group (*P* < 0.05). All other baseline variables showed no statistically significant differences (*P* > 0.05) (**Table 1**).

**Table 1:** Baseline characteristics of the study population.

Parameter	US-guided RFA (n=36)	LA (n=36)	*P*
Sex			0.471
Male	13 (36.1)	16 (44.4)	
Female	23 (63.9)	20 (55.6)	
Age (y)	49.50 (33.25, 57.00)	52.00 (37.50, 59.75)	0.316
Family history of hypertension			0.800
Yes	11 (30.6)	12 (33.3)	
No	25 (69.4)	24 (66.7)	
Antihypertensive drugs			0.151
Yes	18 (50.0)	12 (33.3)	
No	18 (50.0)	24 (66.7)	
Hypertension is pharmaceutically controllable.			0.465
Yes	12 (33.3)	15 (41.7)	
No	24 (66.7)	21 (58.3)	
Cost (¥)	13400.50 (11720.50, 18301.00)	35669.50 (28434.80, 41593.00)	< 0.001

Data are expressed as median and quartiles or numbers of patients (percentages). US-guided RFA, ultrasound-guided radiofrequency ablation; LA, laparoscopic adrenalectomy.

### 3.2 Tumor characteristics

After 1:1 matching, there were no significant differences between the US-guided RFA and LA groups in tumor volume, anatomical location, or ultrasound imaging features (all *P* > 0.05). Additionally, preoperative biochemical indices were assessed, including systolic and diastolic blood pressure, ARR, and serum potassium levels. No statistically significant differences were found between the two groups (all *P* > 0.05). Detailed data are presented in **Table 2**.

**Table 2:** Tumor characteristics in the US-guided RFA and LA groups.

Parameter	US-guided RFA(n=36)	LA(n=36)	*P*
Tumor volume (ml)	1.18 (0.78, 1.73)	1.25 (0.56, 2.50)	0.897
Tumor location			0.056
Left	17 (47.2)	25 (69.4)	
Right	19 (52.8)	11 (30.6)	
Tumor echogenicity			0.710
Hypoechoic	33 (91.7)	31 (86.1)	
Isoechoic	3 (8.3)	5(13.9)	
Hyperechoic	0	0	
Preoperative clinical indicators			
Systolic blood pressure(mmHg)	149.50 ± 26.92	159.97 ± 25.32	0.094
Diastolic blood pressure (mmHg)	93.11 ± 13.47	95.31 ± 14.39	0.506
ARR(ng/dL)/[μg/(L·h)]	52.66 (22.54, 158.91)	29.27 (9.56, 113.33)	0.077
Plasma renin activity [μg/(L·h)]	2.22 (1.17, 8.39)	2.17 (1.73, 3.19)	0.757
Serum K^+^(mmol/L)	3.72 (3.33, 4.13)	3.72 (3.10, 4.28)	0.702

Data are expressed as mean ± SD, median and quartiles or numbers of patients (percentages). US-guided RFA, ultrasound-guided radiofrequency ablation; LA, laparoscopic adrenalectomy.

### 3.3 Intraoperative and postoperative outcomes

Technical success was achieved in all patients in both groups (100%). Compared with the US-guided RFA group, the LA group had significantly longer operative time, greater intraoperative blood loss, and larger incision length (all *P* < 0.001). No recurrence of hypertension or biochemical PA was observed in either group.

There was no significant difference in the overall complication rate between the US-guided RFA and LA groups (5.56% vs. 13.89%; *P* = 0.233). The recorded complications included fever (US-guided RFA, n = 2; LA, n = 4) and bleeding (US-guided RFA, n = 0; LA, n = 1), all of which were classified as Clavien–Dindo grade I. No higher-grade complications were observed. Intraoperative severe hypertension occurred in 10 patients in the US-guided RFA group and 6 patients in the LA group, with no significant difference. Details of intraoperative and postoperative outcomes are summarized in **Table 3**.

**Table 3:** Intraoperative and postoperative outcomes in the US-guided RFA and LA groups.

Parameter	US-guided RFA (n=36)	LA (n=36)	*P*
Procedure time (min)	77.50 (59.25, 104.25)	115.00 (79.25, 140.00)	< 0.001
Length of hospital stay (d)	7.00 (5.25, 8.00)	9.00 (7.00, 16.00)	0.001
Estimated blood loss (mL)	3.00 (2.00, 4.00)	30.00 (16.25, 30.00)	< 0.001
Incision length (mm)	2.00 (2.00,2.00)	13.00 (12.00, 13.00)	< 0.001
Disease progression (hypertension, PA recurrence), n (%)			1.000
Yes	0	0	
No	36	36	
Complications	2(5.56)	5 (13.89)	0.233
Type of complication, n (%)			
Fever	2(5.56)	4 (11.11)	
Bleeding	0	1 (2.78)	
Clavien-Dindo grade, n (%)			
Grade I	2(5.56)	5 (13.89)	
Grade≥II	0	0	
Patients with intraoperative severe hypertension, n (%)			0.257
Yes	10(27.78)	6(16.67)	
No	26(72.22)	30(83.33)	

Data are expressed as median and quartiles or numbers of patients (percentages). US-guided RFA, ultrasound-guided radiofrequency ablation; LA, laparoscopic adrenalectomy.

### 3.4 Blood pressure and biochemical outcomes

**Figure 3:**
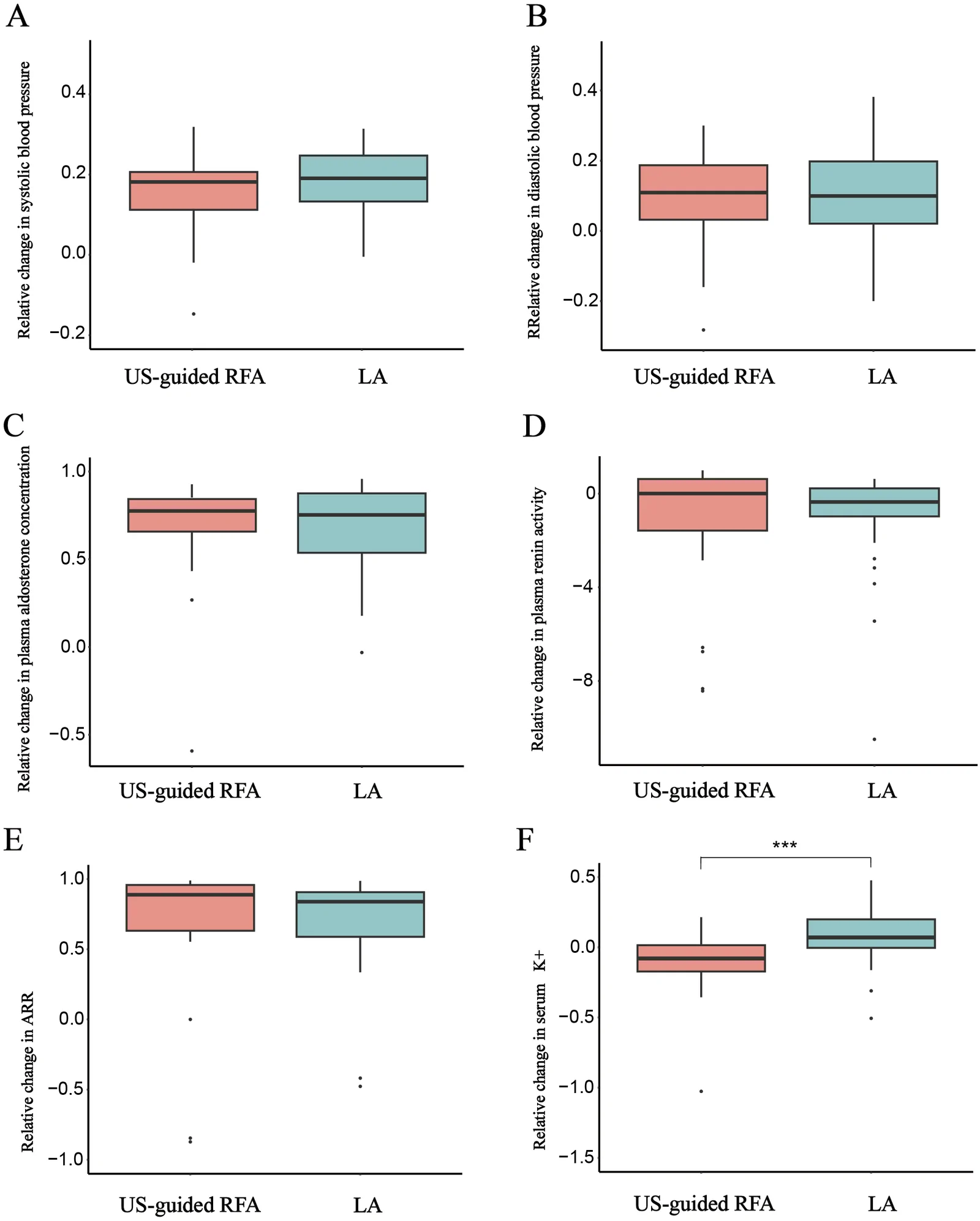
Relative changes in clinical and biochemical parameters in the US-guided RFA and LA groups. **(A)** Relative change in systolic blood pressure. **(B)** Relative change in diastolic blood pressure. **(C)** Relative change in plasma aldosterone concentration. **(D)** Relative change in plasma renin activity. **(E)** Relative change in aldosterone-to-renin ratio (ARR). **(F)** Relative change in serum K^+^. US-guided RFA, Ultrasound-guided Radiofrequency Ablation; LA, Laparoscopic Adrenalectomy; APA, Aldosterone-Producing Adenoma; ARR, Aldosterone-Renin Ratio. ***P < 0.001.

The median follow-up duration was 14 months (IQR, 6–22 months). All patients in both groups successfully completed treatment for APA, and biochemical remission was achieved in all patients. The clinical success rate was 66.67% in the US-guided RFA group and 77.78% in the LA group, with no statistically significant difference (*P* = 0.430). During the follow-up period, none of the patients in the US-guided RFA group underwent a second RFA session or subsequent LA. Detailed blood pressure and laboratory parameter changes are shown in **Table 4** and **Figure 3**.

**Table 4:** Blood pressure control and biochemical changes in the US-guided RFA and LA groups.

Parameter	US-guided RFA (n=36)	LA (n=36)	*P*
Biochemical outcome of PA			1.000
Biochemical remission	36(100)	36(100)	
Persistent biochemical PA	0	0	
Clinical outcome of hypertension			0.430
Clinical success	24(66.67)	28(77.78)	
Absent clinical success	12(33.33)	8(22.22)	
Relative change in systolic blood pressure	0.177 (0.102, 0.205)	0.186 (0.124, 0.248)	0.359
Relative change in diastolic blood pressure	0.11 ± 0.12	0.11 ± 0.13	0.994
Relative change in plasma aldosterone concentration	0.775 (0.662, 0.853)	0.753 (0.525, 0.876)	0.669
Relative change in plasma renin activity	-0.090 (-1.940, 0.669)	-0.354 (-0.990, 0.238)	0.464
Relative change in ARR	0.888 (0.628, 0.961)	0.839 (0.583, 0.917)	0.195
Relative change in serum K^+^	-0.080 (-0.174, 0.016)	0.070 (-0.012, 0.224)	< 0.001

Data are expressed as mean ± SD, median and quartiles or numbers of patients (percentages); Relative change = (pretreatment value − post-treatment value)/pretreatment value. US-guided RFA, ultrasound-guided radiofrequency ablation; LA, laparoscopic adrenalectomy; ARR, aldosterone-renin ratio.

During the follow-up period, there were no significant between-group differences in the relative changes in systolic blood pressure, diastolic blood pressure, plasma aldosterone concentration, plasma renin activity, or ARR (all *P* > 0.05). Serum potassium increased after US-guided RFA but decreased after LA (US-guided RFA vs. LA = −0.080 vs. 0.070; *P* < 0.001).

### 3.5 Intraoperative blood pressure fluctuations

**Figure 4:**
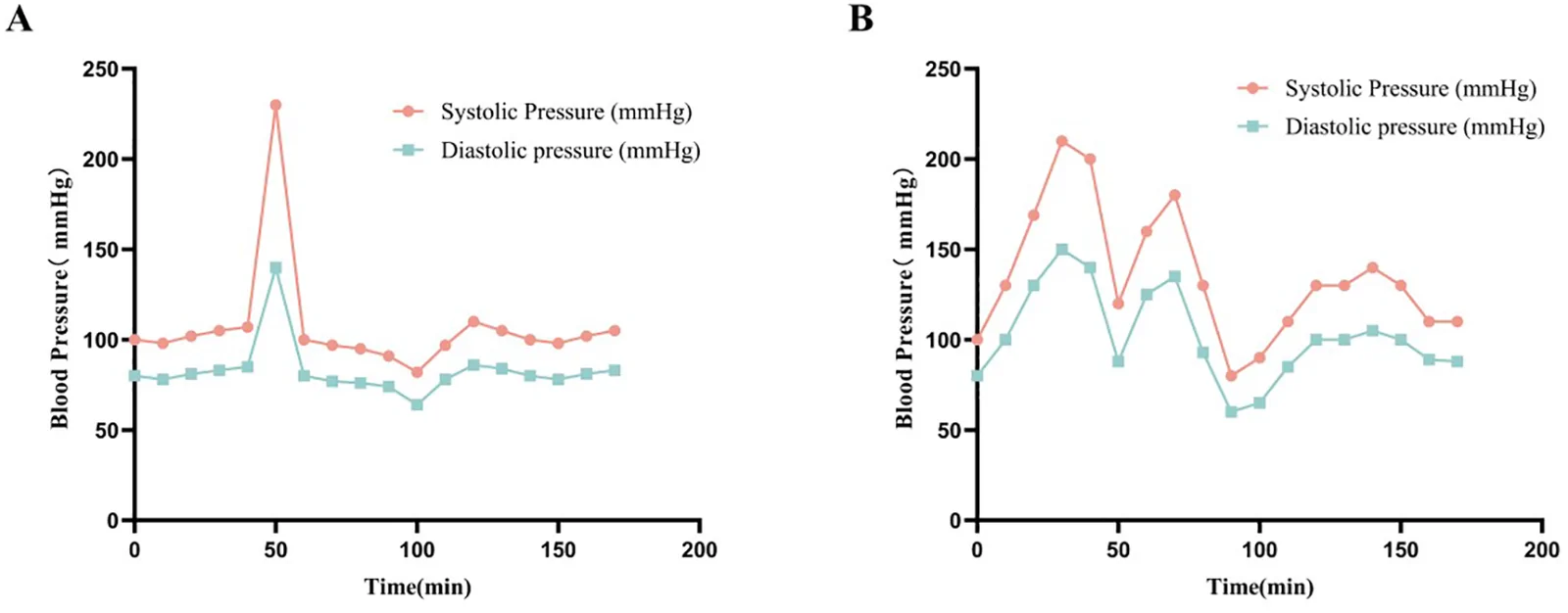
Comparison of intraoperative blood pressure changes between the US-guided RFA group and the LA group. **(A)** In the US-guided RFA group, blood pressure changes were characterized by a rapid but transient elevation. **(B)** In the LA group, blood pressure changes exhibited a slower but more sustained increase. US-guided RFA, Ultrasound-guided Radiofrequency Ablation; LA, Laparoscopic Adrenalectomy.

Among patients who developed intraoperative severe hypertension (US-guided RFA, n = 10; LA, n = 6), intraoperative hemodynamic characteristics were further compared. The number of intraoperative blood pressure elevation episodes was higher in the LA group than in the US-guided RFA group (*P* < 0.001). The time to peak systolic blood pressure, time to blood pressure recovery, and duration of peak systolic blood pressure were also longer in the LA group (all *P* < 0.001). In contrast, the rate of systolic blood pressure elevation was significantly faster in the US-guided RFA group. Detailed intraoperative hemodynamic parameters are presented in **Table 5** and **Figure 4**.

**Table 5:** Intraoperative hemodynamic characteristics among patients with intraoperative severe hypertension.

	US-guided RFA (n=10)	LA (n=6)	*P*
Maximal tumor diameter (mm)	16.40 ± 3.84	19.86 ± 7.71	0.164
Intraoperative start			
Systolic blood pressure (mmHg)	110.10 ± 5.74	112.00 ± 6.87	0.483
Diastolic blood pressure (mmHg)	84.40 ± 3.63	87.21 ± 4.69	0.127
Maximum intraoperative pressure			
Systolic blood pressure (mmHg)	214.70 ± 10.92	194.07 ± 13.15	0.001
Diastolic blood pressure (mmHg)	136.50 ± 3.87	134.86 ± 3.30	0.275
Blood pressure after pharmacological intervention			
Systolic blood pressure (mmHg)	113.00 ± 3.83	112.36 ± 3.52	0.675
Diastolic blood pressure (mmHg)	78.80 ± 5.75	78.43 ± 4.22	0.857
Number of intraoperative blood pressure elevation episodes	1.00 (1.00, 1.25)	2.00 (2.00, 3.00)	< 0.001
Time to peak systolic blood pressure (min)	7.50 (6.00, 9.25)	26.00 (24.00, 29.25)	< 0.001
Time to blood pressure recovery (min)	7.00 (7.00, 8.25)	15.50 (14.00, 18.00)	< 0.001
Duration of peak systolic blood pressure (min)	6.00 (5.00, 8.25)	15.00 (13.00, 16.25)	< 0.001
Rate of systolic blood pressure elevation (mmHg/min)	14.85 (10.38, 17.50)	2.89 (2.56, 3.69)	< 0.001

Data are expressed as mean ± SD, median and quartiles. US-guided RFA, ultrasound-guided radiofrequency ablation; LA, laparoscopic adrenalectomy.

## 4 Discussion

The results of this study demonstrate that there were no significant differences in disease progression rates between ultrasound-guided radiofrequency ablation (US-guided RFA) and laparoscopic adrenalectomy (LA) for the treatment of aldosterone-producing adenoma (APA) (*P* = 1.000). Biochemical remission and blood pressure control outcomes were comparable between the two treatments. However, US-guided RFA had notable advantages. It required a shorter procedural time (77.5 min vs. 115.0 min), resulted in reduced intraoperative blood loss (3 mL vs. 30 mL), and required a smaller incision (2 mm vs. 13 mm) compared with LA (*P* < 0.001).

Recent studies increasingly recognize the benefits of RFA as a minimally invasive alternative to LA in APA treatment (9, 18). While most prior research has focused on CT-guided RFA (19, 20), no studies to date have compared percutaneous US-guided RFA directly with LA. A meta-analysis including five studies reported similar clinical success rates between the two approaches (RFA: 73.1% vs. LA: 68.0%; *P* = 0.20) (21), aligning with our findings (RFA: 66.67% vs. LA: 77.78%). In a study by Liu et al. (22), recurrent hypertension was detected in 6/19 RFA patients and 2/21 LA patients during long-term follow-up (*P* = 0.089). In contrast, no recurrence of hypertension or biochemical PA was observed in our study during a median follow-up of 14 months (IQR, 6–22 months). The favorable outcomes in our cohort may partly reflect the integration of AVS and/or CXCR4-targeted imaging with cross-sectional and ultrasound findings for target selection. In addition, real-time ultrasound allowed multiplanar lesion localization and dynamic adjustment of the needle trajectory, while CEUS enabled immediate assessment of residual perfusion and the ablation margin; fusion navigation was also used in selected technically challenging cases. These factors may have contributed to more accurate targeting and complete ablation. Nevertheless, the longer follow-up in the previous study should be considered when comparing recurrence outcomes.

A meta-analysis by Chen and Guo et al. (21, 23) concluded that RFA yielded greater reductions in systolic and diastolic blood pressure compared to LA. However, in our cohort, systolic blood pressure reduction was slightly lower in the RFA group (0.177 vs. 0.186; *P* = 0.359), and diastolic reduction was similar (0.11 vs. 0.11; *P* = 0.994). This discrepancy may be attributed to the limited sample size, potentially underpowering the detection of intergroup differences. Nevertheless, both modalities achieved comparable rates of blood pressure control.

Although the minor complication rate was lower in the US-guided RFA group (5.56% vs. 13.89%; *P* = 0.233), the difference was not statistically significant. This favorable trend may result from preoperative planning using both CT and CEUS for precise pathway design. In patients with anatomically complex lesions, intraoperative fusion navigation systems enhanced localization accuracy and visualization of ablation margins, thereby minimizing complications (24). Experimental evidence suggests that the fat tissue surrounding the adrenal gland may help limit thermal spread to adjacent structures (25). The combined use of hydrodissection techniques further reduces heat spread and protects neighboring organs (26).

The incidence of intraoperative severe hypertension did not significantly differ between groups (27.78% for US-guided RFA vs. 16.67% for LA; *P* = 0.257). However, their patterns varied: US-guided RFA was associated with a rapid but brief increase in blood pressure, while LA induced a slower and more sustained elevation. This variation may reflect differences in how each modality affects the tumor and surrounding tissue. RFA induces abrupt functional changes through localized thermal effects (27). In contrast, LA leads to gradual changes due to wider tissue resection and longer operative time (28). These findings suggest that US-guided RFA may reduce the frequency and duration of episodes of intraoperative severe hypertension. However, intraoperative peak systolic pressure was significantly higher with US-guided RFA, warranting caution due to potential acute risks. Future large-scale prospective studies are needed to optimize safety and manage hypertensive fluctuations in both approaches.

Limitations of this study include the small sample size and short follow-up duration. Despite multimodal assessment, an imaging-occult APA could not be completely excluded. Although biochemical and blood pressure control were comparable between groups, the long-term efficacy and safety of RFA require further investigation in larger studies with longer follow-up.

In conclusion, US-guided RFA and LA demonstrated comparable short-term outcomes for unilateral APA. US-guided RFA appears to be a safe and effective minimally invasive alternative. Further research is warranted to assess its long-term utility and broaden treatment options for APA patients.

## Data Availability

The raw data supporting the conclusions of this article will be made available by the authors, without undue reservation.
